# Gyrocardiography: A Review of the Definition, History, Waveform Description, and Applications

**DOI:** 10.3390/s20226675

**Published:** 2020-11-22

**Authors:** Szymon Sieciński, Paweł S. Kostka, Ewaryst J. Tkacz

**Affiliations:** Department of Biosensors and Processing of Biomedical Signals, Faculty of Biomedical Engineering, Silesian University of Technology, Roosevelta 40, 41-800 Zabrze, Poland; pkostka@polsl.pl (P.S.K.); etkacz@polsl.pl (E.J.T.)

**Keywords:** gyrocardiography, cardiac vibrations, waveform description, cardiovascular diseases, signal processing, health monitoring

## Abstract

Gyrocardiography (GCG) is a non-invasive technique of analyzing cardiac vibrations by a MEMS (microelectromechanical system) gyroscope placed on a chest wall. Although its history is short in comparison with seismocardiography (SCG) and electrocardiography (ECG), GCG becomes a technique which may provide additional insight into the mechanical aspects of the cardiac cycle. In this review, we describe the summary of the history, definition, measurements, waveform description and applications of gyrocardiography. The review was conducted on about 55 works analyzed between November 2016 and September 2020. The aim of this literature review was to summarize the current state of knowledge in gyrocardiography, especially the definition, waveform description, the physiological and physical sources of the signal and its applications. Based on the analyzed works, we present the definition of GCG as a technique for registration and analysis of rotational component of local cardiac vibrations, waveform annotation, several applications of the gyrocardiography, including, heart rate estimation, heart rate variability analysis, hemodynamics analysis, and classification of various cardiac diseases.

## 1. Introduction

The human heart is an organ located between the lungs, in the middle compartment of the chest [1] which pumps the blood through the blood vessels of the cardiovascular system [2]. The heart undergoes repeating changes in different directions and orientations related to the cardiac cycle [3]. In each cardiac cycle the contraction of helically oriented muscle fibers result in a coordinated wringing motion to the myocardium [4] which plays an important role in systolic and diastolic myocardial deformation [4,5,6,7].

For instance, the movement of the base of the left ventricle indicates the ventricular systolic and diastolic function. In physiological conditions the base of the heart moves towards the apex [5,8]. Mechanical compressions of the heart that generate low frequency vibrations originate in action potentials induced by the sinoatrial node [9]. These vibrations diffuse through the chest and can be detected at the sternum by accelerometers and gyroscopes [9,10].

Studies on the cardiac mechanical activity have been conducted over the years using both invasive and non-invasive techniques in animals and humans [11]. In 1975, Ingels et al. evaluated left ventricle performance in 24 patients based on multiple implanted radiopaque markers and cardiac fluoroscopy [12]. Other non-invasive approaches to study the dynamics of cardiac motion and myocardial tissue function were based on optical devices [13,14] tagged magnetic resonance imaging (tagged MRI) [15], tissue Doppler imaging (TDI) [16] and speckle tracking imaging [17].

The first studies on the feasibility of heart monitoring using wearable devices were performed using accelerometer and gyroscope sensors built in Google glasses, wrist worn devices, smart phones, and chest worn patches [18,19,20,21]. New microelectronics and signal processing technologies have provided unprecedented opportunities to reintroduce some of cardiac vibration registration techniques as useful tools in the diagnosis of cardiovascular system [22].

Cardiac vibrations signals have been investigated over the past century to determine their relationship to the cardiac cycle and find the potential use in non-invasive cardiology [22]. The non-invasive methods of cardiac mechanical monitoring known under the umbrella term “mechanocardiography” (MCG) are ballistocardiography (BCG), seismocardiography (SCG) and gyrocardiography (GCG) [22,23,24,25,26].

Ballistocardiography (BCG) is the recording of the reactionary forces of the body invented by Gordon in 1877 [23]. BCG measures the whole body recoil or ballistic forces in response to the blood ejection from aorta into the vascular tree [27,28]. According to the sophisticated in vivo experimental examinations and a complementary mathematical model, BCG waves are formed due to blood pressure gradients in the ascending and descending aorta [29].

Seismocardiography is the recording of cardiac vibrations reflected as the three-dimensional (3D) linear acceleration on the chest wall [9] invented independently by Mounsey [30] in 1957 as the “praecordial ballistocardiography” and Bozhenko in 1961 [25]. The name “seismocardiography” was coined by R. Baevskii et al. in 1964 as the combination of “seismo” and “cardiography”, which describe the measurement of cardiac vibrations on the chest [31].

Gyrocardiography (GCG) is a technique for recording of three-dimensional angular velocity and displacement of the thorax associated with cardiac activity using a gyroscope placed on a chest wall [8,11,32]. This technique was invented by Meriheinä et al. in 2015 [32], and its name was proposed by Tadi et al. in 2016 as the blend of “gyroscope” and “cardiography” [8,33]. The gyroscope is an active sensor which measures its own angular velocity (gyration) and can detect very small angular displacements caused by the cardiac activity [8,32]. Today, the preferred type of gyroscopes is MEMS (microelectromechanical system) gyroscope due to its size, cost, power consumption and accuracy [8,11]. An example of such gyroscope is a low power 3-axis MAX21000 gyroscope (Maxim Integrated, San Jose, CA, USA) which was used in [8,11]. Its measurement range is ± 250 degrees per second (dps, °/s), the noise density is 9 mdps/$\sqrt{Hz}$ and the sensitivity is 120 digits/dps for the measurement range of ± 250 dps [34].

Although the BCG, SCG, and GCG register the mechanical activity of the cardiovascular system in the cardiac cycle, these techniques represent different aspects of such analysis. Ballistocardiography measures the recoil forces of the whole body in response to the cardiac activity [22,24], whereas the SCG and GCG measure local vibrations of the chest in response to the cardiac cycle [8,22,25]; seismocardiogram (SCG signal) represents the linear component of precordial vibrations [27] and gyrocardiogram (GCG signal) represents the rotational component of precordial vibrations [8].

SCG and GCG signals are mutually orthogonal and they are susceptible to different noise characteristics, which may help analyze various aspects of cardiac mechanical activity [9]. Up to 60% of cardiac vibrational energy is contained in the gyration signal [21], which suggests that the gyrocardiographic signal has higher noise rejection ratio than the seismocardiographic signal [11,35]. GCG may provide novel insights into cardiac fiducial points [11,35], higher fidelity for certain types of motion artifact [35,36,37,38], and can assist in SCG heartbeat detection using kinetic energy envelopes [38,39].

**Figure 1:**
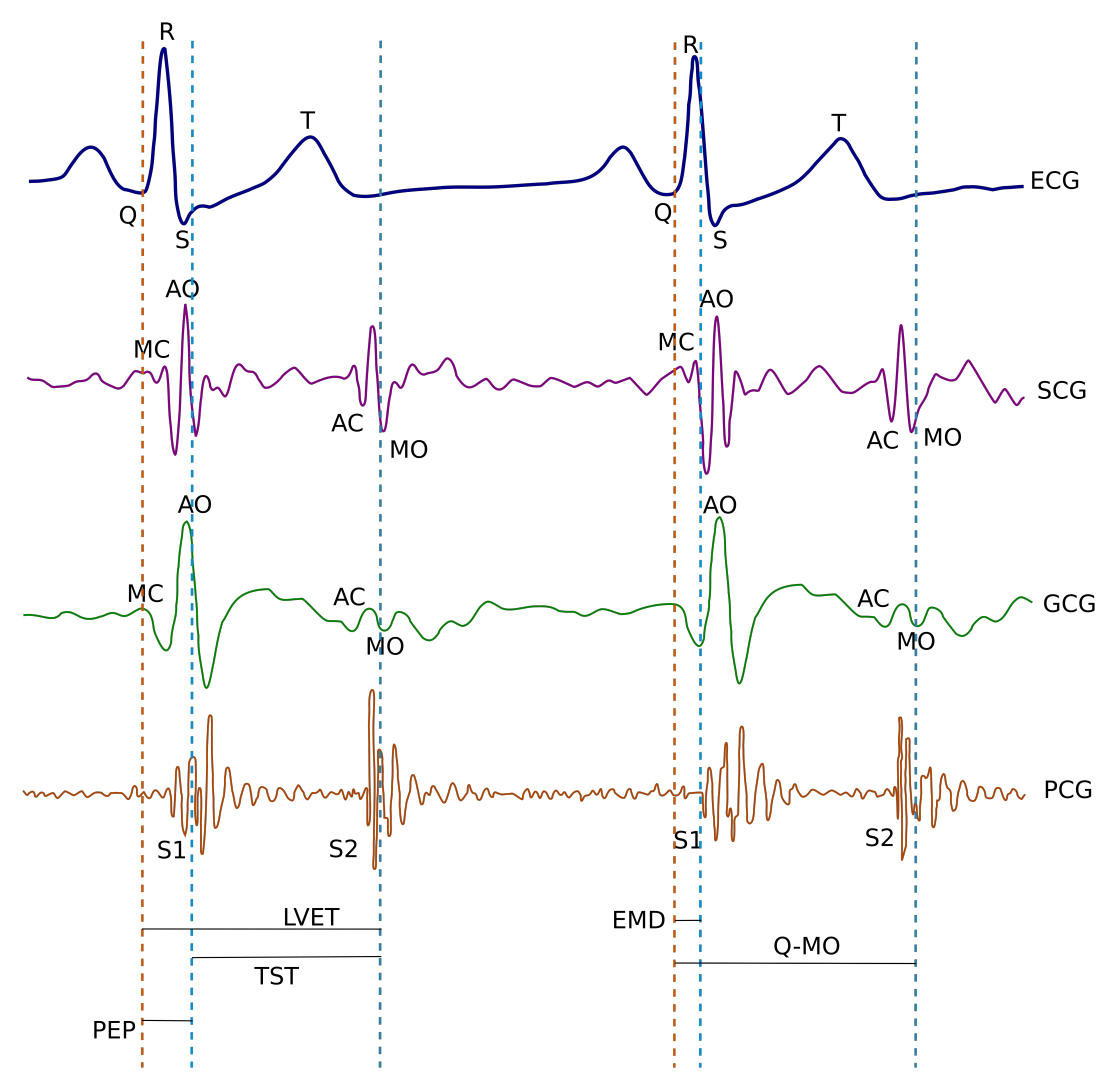
Simultaneous sample recordings of ECG, SCG (z-axis), GCG (y-axis), and phonocardiographic (PCG) signals captured in the supine position. The MC and MO waves in SCG and GCG correspond to mitral valve closure and opening; the AC and AO waves in SCG and GCG corresponded to the aortic valve closure and opening. S1 and S2 waves on PCG corresponded to mitral and aortic valve closure, respectively. EMD (electromechanical delay), PEP (Pre-ejection period), TST (total systolic time) and LVET (left ventricular ejection time) and systolic time intervals are also illustrated. The diagram is a modified version of the diagram originally published in the article by Dehkordi et al. in [42] with a GCG y-axis waveform derived from a diagram published in the work by Tadi et al. [11]. The cited works are available under the license CC-BY 4.0.

The model describing the relationship between sternal vibrations and the deformation of cardiac walls is still an open issue [11,40]. However, key events in the cardiac cycle show a clear correlation with the features in the SCG and GCG waveforms [8,9] (see Figure 1). The electromechanical delay (EMD) which occurs in the cardiac cycle observed in the analysis of simultaneous recordings of electrocardiograms and cardiac vibrations may bring new insights into the assessment of myocardial function [41]. Therefore, wearable/mobile GCG may be useful in quantification of beat-by-beat dynamics of cardiac time intervals and can potentially provide the insight into the hemodynamic variables and myocardial contractility [11].

To the best of the authors’ knowledge, available reviews of the current state of the art in the analysis of cardiac vibrations provide only brief mentions of gyrocardiography. The purpose of this work is to provide the comprehensive literature review of the current state of the knowledge of gyrocardiography and to summarize the history, definition, waveform description and applications of this technique, and also identify potential gaps in the knowledge of gyrocardiography.

## 2. Materials and Methods

### 2.1. Search Strategy

The search of scientific papers (journal articles, conference proceedings, patents) on gyrocardiography was conducted between November 2016 and September 2020 using Google Scholar, IEEEXplore, Scopus, Web of Science Core Collection and PubMed for the following expressions: “gyrocardiography”, “gyrocardiographic”, “gyrocardiogram”, “gyrocardiograms”, “GCG”, “mechanocardiography”. More resources were retrieved after reviewing the reference section of the initial search results. The total number of analyzed papers was about 55.

### 2.2. Inclusion and Exclusion Criteria

The criteria for inclusion of a paper in the review are: (1) written in English; (2) providing a definition, waveform description or medical background of gyrocardiography or (3) describing an application of gyrocardiography (GCG) or (4) describing the analysis of GCG waveforms. Articles which do not include the use or description of gyrocardiography in any form were excluded from the literature review.

## 3. Results

### 3.1. History

#### 3.1.1. Before 2015

The cardiac mechanical activity has been studied over the years using both invasive and non-invasive techniques in animals and humans [11]. The first study related to gyrocardiography was the evaluation of left ventricle performance in 24 patients based on multiple implanted radiopaque markers and cardiac fluoroscopy by Ingels et al. in 1975 [12]. Other non-invasive approaches to investigate the cardiac motion and myocardial function included optical devices [13,14], tagged magnetic resonance imaging (tagged MRI) [15], tissue Doppler imaging (TDI) [16] and speckle tracking imaging [17].

Invasive techniques for assessment of the left ventricular function in animals based on implantable gyroscopes and accelerometers were introduced by Marcelli et al. [6,43], Hyler et al. [44], and Grymyr et al. [45]. The findings of [6,43,44,45] reported promising results which might suggest the development of implantable devices for the continuous monitoring of cardiac function [11].

#### 3.1.2. 2015–2016

Gyrocardiography was first described in 2016; however, the first description of this technique was then called “angular ballistocardiography” and was published in 2015 in the patent number WIPO 2015/036925 A1 by U. Meriheinä et al. [32]. The invention described in the patent is a heart monitoring system that uses a sensor of angular motion placed to the chest wall, near the heart, to obtain a signal named “angular ballistograph”. The obtained signal indicates the rotational movement of a chest related to the cardiac activity [32]. The heart monitoring system was designed to provide additional support of other physiological signals, such as photoplethysmograms, electrocardiograms or magnetic signals measurement. The proposed applications include heart rate measurements, the diagnosis of atrial fibrillation (AFib) and other cardiac abnormalities [32].

The study conducted by Jia et al. [20] described the method for estimation of heart rate from the acceleration and gyration signals related to cardiac cycle which were acquired on the chest wall. Based on the nature of the analyzed signals, we may treat them as acquired signals as seismocardiograms and gyrocardiograms based on the definition in [8].

In 2016, M. J. Tadi et al. presented gyrocardiography as “a non-invasive technique based on a tri-axial gyroscope sensor (preferably MEMS) which measures angular velocities of the chest as a response to the motion of the heart” [8]. Because the definition of gyrocardiography in [8] was based on [32], we can treat “angular ballistocardiography” and “gyrocardiogram” are synonyms. The works published since 2016 use consequently the terms “gyrocardiography” for the method and “gyrocardiogram” for the signal. In the same year Tadi et al. published papers on the applications of GCG in PET (positron emission tomography) gating [33,46] and Lahdenoja et al. published the first study on heart rate variability (HRV) analysis on gyrocardiograms [47].

#### 3.1.3. 2017

In 2017, C. Yang and N. Tavassolian [35,39,48] and Tadi et al. [11] published more comprehensive evaluations of gyrocardiograms; Yang and Tavassolian in [48] proved that SCG or GCG signals in combination with photoplethysmography (PPG) can be used to measure pulse transit time (PTT) and may be performed using a smartphone and PPG sensor connected to the audio input. Tadi et al. in the study on nine health volunteers provided a comprehensive comparison of the gyrocardiography with electrocardiography (ECG), echocardiography, pulse wave Doppler (PWD), the waveform annotation and estimation of hemodynamic variables [11]. They confirmed very high correlation of heart rate obtained with GCG and ECG, left ventricular ejection time (LVET) and pre-ejection period (PEP). These findings were also proved by Yang and Tavassolian in [35]. Another important conclusion is the fact that the morphology of GCG signal is consistent between different gyroscopes and the difference in the signal amplitude is within a fer degrees per second [11]. T. Hurnanen et al. proposed an algorithm for heartbeat detection in SCG and GCG signal which confirmed the reliability of heart monitoring based on gyrocardiography and seismocardiography [49], which is in line with the findings in [11].

In the same year Migeotte and Delière [50] published the patent on the improvements related to heart monitoring. The main improvement was the use of six-axis inertial measurement units in order to register linear and rotational components of cardiac vibrations. Tuominen et al. in [51] described the smallest device capable of monitoring and recording electrical and mechanical activity of the heart (50 mm length, 32 mm width, 11.9 mm height with mechanics). Lahdenoja et al. [52] and Tadi et al. [53] described the methods for assessing the quality of SCG and GCG signals based on one-dimensional local binary patterns (1D LBP) in combination with support vector machine (SVM) [52] and kernel support vector machine (KSVM) [53].

In the article published in 2017 Yang, Tang and Tavassolian proved that the combination of SCG and GCG can be used to automatic annotation of SCG signals [39]. Another works on the applications of gyrocardiography in the diagnosis of cardiovascular diseases published in 2017 are: the work by Hurnanen et al. on the detection of atrial fibrillation based on seismocardiograms and gyrocardiograms [49] and the work [54] on the detection of myocardial infarction.

#### 3.1.4. 2018

The works on gyrocardiography published in 2018 describe detecting various cardiovascular diseases [55,56], motion noise cancellation [36], standalone heartbeat detection [37,57,58] and the measurement of pulse transit time [59]. The works of Iftikhar et al. [55], Mehrang et al. [60] and Yang et al. [56] were the first attempts to use gyrocardiographic data to classify various cardiac diseases (myocardial infarction and coronary artery disease) using support vector machine (SVM) [60], kernel-based SVM (KSVM) and random forests (RF) [55].

The detected cardiovascular diseases include atrial fibrillation [55,61], myocardial infarction [55,60] and coronary artery disease (CAD) [55]. The classification of diseases was based on machine learning, namely SVM with leave-one-out cross-validation (LOOCV) [60], KSVM and random forests (RF) with or without majority voting [55].

In 2018 Vega-Martínez et al. published a review article on the heart rate measurements mentions the use of gyrocardiography as a method for monitoring the heart rate which may be used to perform HRV analysis [62]. Standalone heart beat detection methods published in 2018 include the methods proposed by Lee et al. [37,38], Hernandez and Cretu [57] and Kaisti et al. [58] with complementary data set “Mechanocardiograms with ECG Reference” published on IEEE DataPort [63].

In Ref [36] Yang and Tavassolian underlined that the motion artifacts hamper the implementation of cardiac mechanical signals in clinical scenarios [27,37]. Therefore, they proposed and evaluated the method for removing motion artifacts by constrained independent component analysis (ICA). The results of this approach suggest that the proposed approach can improve the accuracy of the heart rate and PEP measurement.

#### 3.1.5. 2019

In 2019 Casenella et al. published a review of the current state of knowledge in cardiac mechanical signals. The signals can be divided into two main categories: whole-body signals and local pulses. Whole-body signals are represented by ballistocardiography. Local pulses have two categories: audible signals (phonocardiograms) and infrasound. The phonocardiogram (PCG) has a long history since its discovery in 1908 and is a signal which can be recorded during the cardiac auscultation, one of the most common medical procedures [22]. Gyrocardiography is defined in [22] as a local pulses signal, obtained by placing a gyroscope in the place of an accelerometer in seismocardiography [8]. The works published in 2019 on gyrocariography describe new topics and already known topics, such as the heart beat detection [9], detection of atrial fibrillation [64,65], hemodynamics analysis [42,66,67], pulse transit time measurement [68] and respiratory and cardiac gating [69]. The review of the state of seismocardiography by A. Taebi et al. in 2019 reveals that the analysis of rotational vibrations may provide complementary information to the SCG signal analysis [70].

New topics on gyrocardiography described in 2019 include performing cardiac monitoring of dogs via smarthphones [71], fetal heart rate (FHR) monitoring [72], investigating the influence of respiratory volume on the SCG and GCG waveforms [73] and evaluating the motion-based heart rate measurement [74]. Yang et al. conducted the study on FHR estimation based on fetal cardiotocography sensor and the reported positive percent agreement (the equivalent of sensitivity/TPR when the reference is uncertain [75]) was 75.52% from GCG [72]. Based on the findings in [73], the variation in the signal increases when inhaling. The evaluation of the motion sensors offers an opportunity to track the behavior and heart rate in workplace using econsumer electronics devices [74].

#### 3.1.6. 2020

In 2020 Achi’ldiev et al. provided an extended overview and comparison of the gyrocardiograms to seismocardiograms and electrocardiograms. They compared the waveforms, spectra, amplitude ranges, bispectra, the length and the area of the cardiac cycle [76]. In the same year, Mehrang et al. [77] proposed a new classifier of cardiac diseases (atrial fibrillation and acute decompensated heart failure) based on SCG and GCG signals. The classification was based on random forests, extreme gradient boosting (XGB) and logistic regression (LR). The best performing classifier was XGB for atrial fibrillation and LR for acute decompensated heart failure.

Yang et al. [78] proposed a machine learning-based method for classification of aortic stenosis. Another studies describe the estimation of static lung volume states [79]. Based on the findings of the study of Yang et al. [78], the patients after TAVR are not recognized as healthy people because artificial heart valves produce different vibrations than natural valves.

In this year Clairmonte et al. in [79] confirmed the feasibility of classification of two lung volume states (high and low volume state) on 50 participants. D’Mello et al. in [80] identified the heart sounds based on seismocardiography and gyrocardiography with a high correlation coefficients of 0.9887 for HR measured with concurrent ECG measurement. Another study on the heart rate estimation in 2020 was conducted by Ezz Aboulezz et al. [81]. In the same year Ezz Aboulezz et al. [81] developed the beat detector based on Autocorrelated Differential Algorithm (ADA), which uses the autocorrelation of the signal to calculate the HR [9,82].

Two works of Siecinski et al. [83,84] describe the HRV analysis on gyrocardiograms as the comparicon of HRV indices, based on the approach in [85,86,87]. The HRV analysis in [83,84] was performed on 29 recordings of GCG signals from [63] described further in [58] in time domain and frequency domain. The results indicate that the HRV analysis based on the GCG can be considered reliable for healthy subjects. However, the HRV analysis in various cardiac diseases requires further research [83].

### 3.2. Number of Works on Gyrocardiography

To present the changes in the number of published works on gyrocardiography, the following databases and search engines were examined: Google Scholar, Scopus, PubMed, IEEEXplore, Web of Science Core Collection and Springer Link. The number of works published in each year and the overall number of works on the topic was obtained for the search query “gyrocardiogram OR gyrocardiography OR gyrocardiograms”. The results shown in Table 1 includes all the works on gyrocardiography available in each considered database or search engine separately.

### 3.3. The Definition and Signal Characteristics

Based on the analyzed works the definition of gyrocardiography can be formulated as:

a non-invasive technique based on a tri-axial gyroscope sensor (preferably MEMS) which measures three-dimensional angular velocities of the chest as a response to the motion of the heart [8,9,11,32].

The signal obtained in gyrocardiography is named *gyrocardiogram* [8]. Gyrocardiogram is a low frequency mechanical signal classified by Casanella et al. as a local pulse signal [22] measured in degrees per second (dps, °/s) and its frequency range is 1–20 Hz [8]. Its amplitude range is within a few dps [11]. The GCG along with the SCG and BCG constitute the mechanocardiography (MCG) or vibrational cardiography (VCG). The first term was proposed by [24,49,55,58,60,64,65,71,73,77,88] and the second term was proposed by [9,73,79,80].

#### 3.3.1. Signal Registration

**Figure 2:**
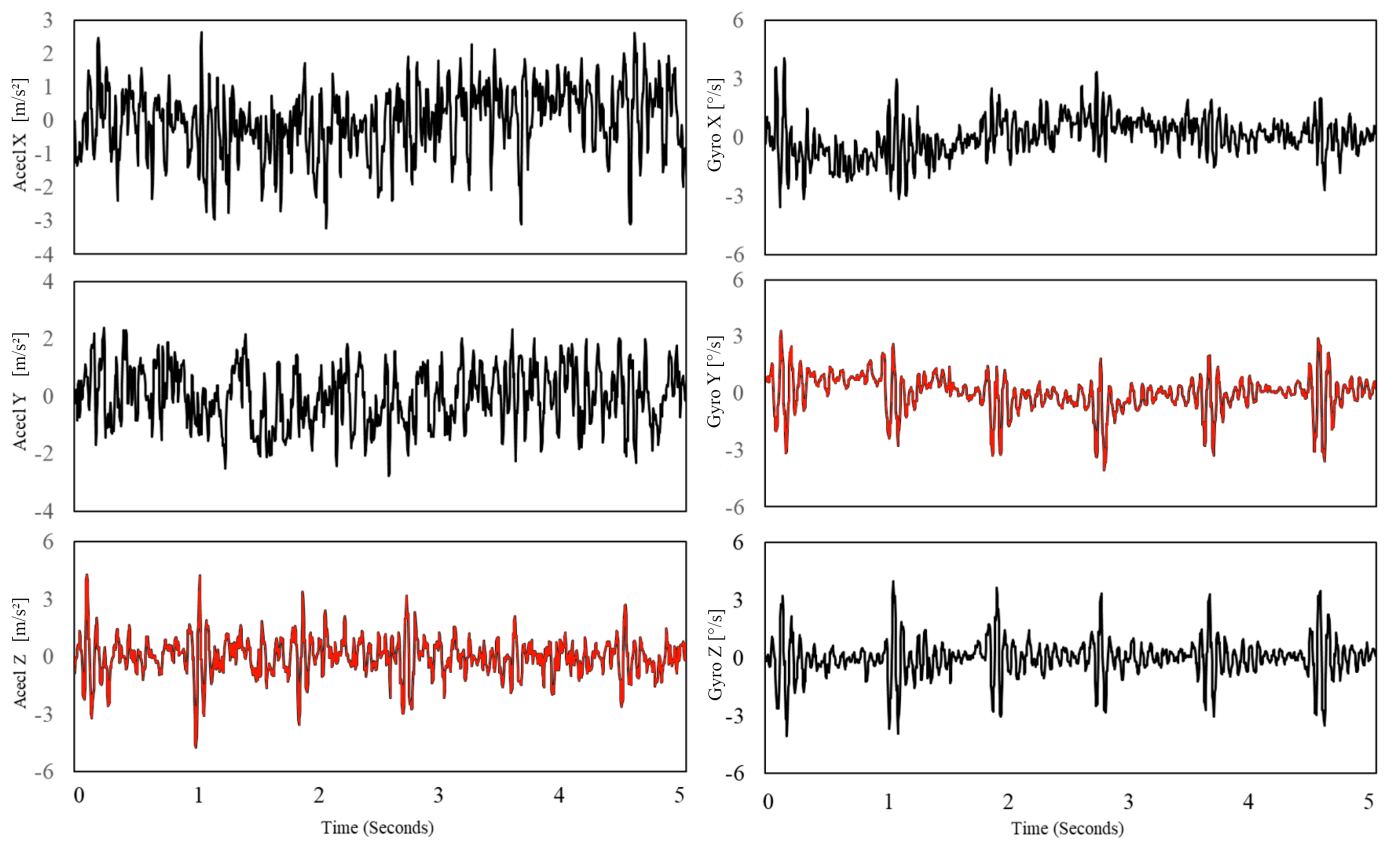
Raw SCG signal in three axes (**left**) and three orthogonal components of raw GCG signal (**right**). Typical axes of seismocardiogram (z-axis) and gyrocardiogram (y-axis) are shown as a red line. Modified version of the graph derived from the article [37] by Lee et al. under the license CC-BY 4.0.

**Figure 3:**
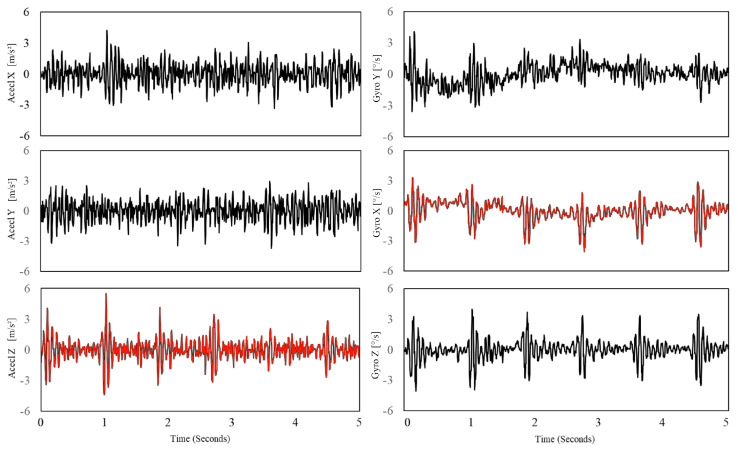
SCG signal (**left**) and GCG signal (**right**) in three axes after applying Savitzky-Golay filter to remove motion artifacts. Typical axes of seismocardiogram (z-axis) and gyrocardiogram (y-axis) are shown as a red line. Modified version of the graph derived from the article [37] by Lee et al. under the license CC-BY 4.0.

The gyrocardiogram is registered in one or three axes. Each axis has a distinctive signal pattern, with a magnitude of a few degrees per second (dps or °/s) [8,11]. For human subjects, the x-axis is the horizontal axis, the y-axis is defined as the vertical axis and the z-axis is the dorso-ventral axis [9]. The GCG signals in x- and y axes are usually of better quality [8,11]. Gyrocardiograms registered in one axis are usually the signals in y-axis because of the high signal-to-noise (SNR) ratio [8,11,57,58,61]. Figure 2 shows raw SCG and GCG signal waveforms in three orthogonal x, y and z axes and Figure 3 presents 3-axial SCG and GCG signals after applying Savitzky-Golay filter to remove motion artifacts.

To ensure high quality of the registered signal, the sensor (gyroscope) should be placed as close to the heart as possible [32]. Because the GCG and SCG are mutually orthogonal, the sensor can be placed at the xiphoid process of the sternum [26].

**Figure 4:**
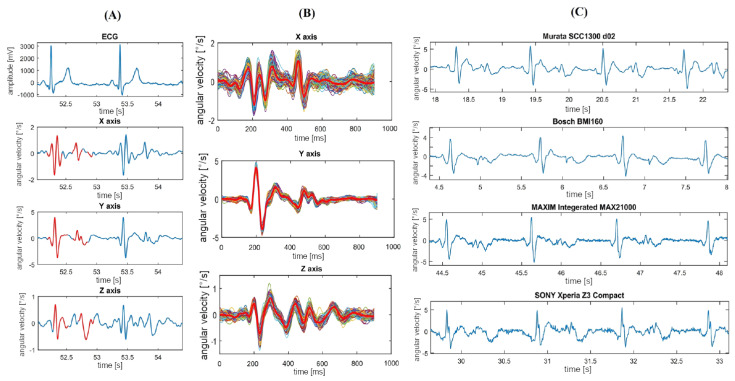
(**A**) Typical GCG waveforms in the x, y, and z axis and reference ECG signal. (**B**) 3-axis ensemble averaged GCG signals. (**C**) GCG waveforms in the y-axis obtained using different sensors (Murata SCC1300d02, Bosch BMI 160, Maxim Integrated MAX21000, and the SONY Xperia Z3 compact). The graph derived from the article [11] by Tadi et al. under the license CC-BY 4.0.

**Figure 5:**
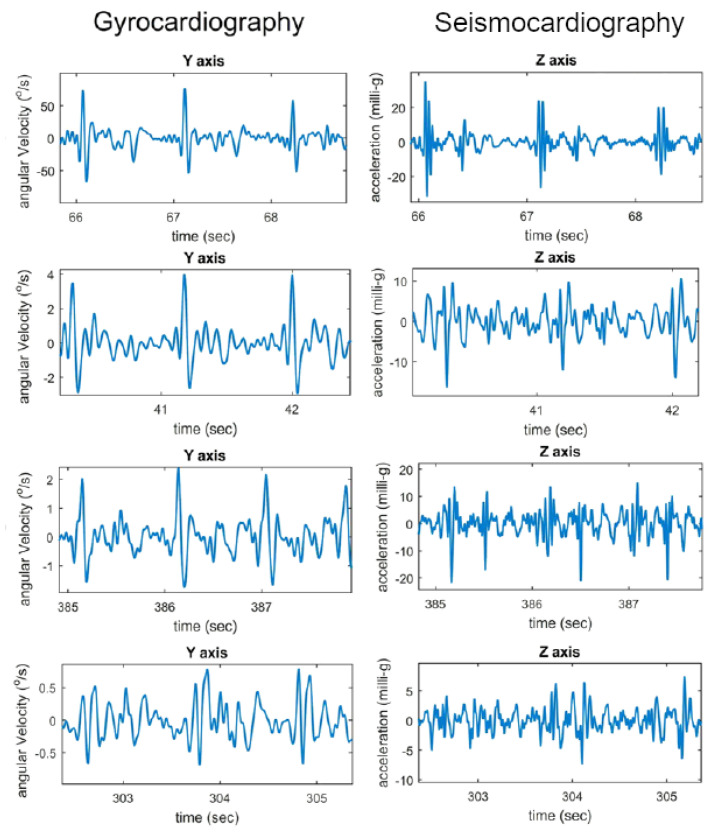
An example of GCG y-axis and SCG z-axis inter-subject variation with various levels of signal quality (top to bottom: good, medium, low and very low). Adapted version of the graph derived from the article [11] by Tadi et al. under the license CC-BY 4.0.

The morphology of the gyrocardiogram is similar in shape in different subjects, despite using different gyroscopes with various technical specifications (overall quality of signal, noise level, power consumption, full scale, range) [11]. An example of GCG signals acquired with the different gyroscopes (Murata SCC1300d02, Bosch BMI 160, Maxim Integrated MAX21000, and the SONY Xperia Z3 compact) is shown in Figure 4. Unfornunately, there is no clear indication whether the signals were acquired on one or more subjects involved in preparing the comparison. The inter-subject variability of GCG y-axis and SCG z-axis signals at various quality levels (top to bottom: good, medium, low and very low) are shown in Figure 5. Despite the decrease of quality shown top to bottom, the GCG y-axis signal morphology remains stationary in comparison with the SCG z-axis signal.

#### 3.3.2. The physics of Gyrocardiography

Gyrocardiography is the measurement of cardiac vibrations with a gyroscope placed on the chest wall [8,11,32]. The gyroscope measures its own angular velocity and is capable of accurate detection of cardiac vibrations on sternum [8,32]. These vibrations are caused by the twisting and untwisting motion of the myocardium [8].

The motion of an object has six degrees of freedom: translation in three orthogonal axes and rotations in three orthogonal axes. Translations are measured by an accelerometer and rotations are measured by a gyroscope. The measurement of angular velocity in MEMS sensors is based on exploiting the Coriolis force [32,89]. Due to this fact, the gyrocardiograms are less affected by the changes of the posture of the subject than the seismocardiograms [32].

Based on the model presented in [8], the relationship between an angle of rotation and cardiac tissue velocity can be expressed as the approximation of the rotation of the gyroscope in the horizontal plane by angle α by measuring the heights of the edges of the gyroscope (denoted as *a* and *b*) at time *t* [8]. The approximation expressed in Equation (1) is true as long as α is small ([90], p. 30–32).

(1) α≈sinα=Δh(t)/d,

where $\Delta h=h_{a}\left(t\right)-h_{b}\left(t\right)$, and *d* is the width of the gyroscope [8].

**Figure 6:**
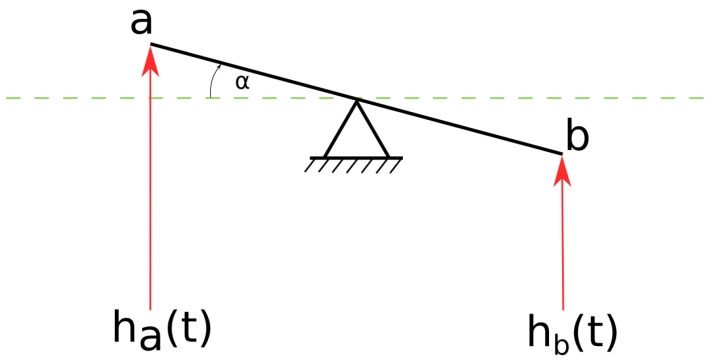
The model of the relationship between the rotation of the horizontal plane α and angular velocity ω.

The angular velocity (gyration) ω measured by the gyroscope can be expressed as:

(2) $$\omega \approx \frac{d}{\mathrm{dt}}\Delta h\left(t\right)/d=\frac{v_{a}\left(t\right)-v_{b}\left(t\right)}{d},$$

where $v_{a}\left(t\right)$ and $v_{b}\left(t\right)$ are the vertical velocities of *a* and *b* points shown in Figure 6 and *d* is the width of the gyroscope.

This mathematical model states that we can interpret the angular velocity (gyration) as a differential velocity corresponding to the edges of the sensor. In other words, the GCG evaluates the motion of the chest at the edges of the gyroscope. If the motions originate from two different points in the heart, the result is related to the strain rate between these points [8].

#### 3.3.3. Physiological Sources of Gyrocardiography

Cardiac vibrations detected at the sternum are induced by the action potentials recorded by ECG [9]. In each cardiac cycle, the contraction of helically oriented muscle fibers causes a coordinated wringing motion of the heart [4]. The longitudinal retraction of the myocardium causes the move of the left ventricle towards the apex. Linear contribution of the muscle fibers contraction in the long axis of the heart indicates ventricular systolic and diastolic mechanical function [5,11,91].

Gyrocardiography registers rotational components of the precordial vibrations in response to myocardium movement [11]. Because the waves in the GCG and SCG signals correspond to the same physiological events visible in cardiac vibrations [40,76,92], we can treat the SCG and GCG as complimentary techniques [8,47].

Cardiac vibrations registered in SCG and GCG contain are probably the result of the cardiac mechanical processes, including myocardial contraction, valve movement, blood flow turbulence and changes in momentum [70]. The characteristics of cardiac vibrations reflect physiologic [40] and pathologic processes [93].

The relationship between SCG waves and cardiac activity is still not fully understood because of the waveform variations in different studies and lack of understanding of the exact sources of SCG waves [70]. However, according to the studies on the relationship between the cardiac cycle and registered vibrations [8,10,11,33,94] and simultaneous recordings of SCG, ECG and echocardiograms [8,10,11,33,94], the SCG signal contains the peaks and valleys of the SCG in the cardiac cycle corresponding to mitral valve opening (MO) and closure (MC), isovolumetric contraction (IVC), rapid ejection (RE), aortic valve opening (AO), closure (AC) [27,95], and also systolic and diastolic velocity [11].

**Figure 7:**
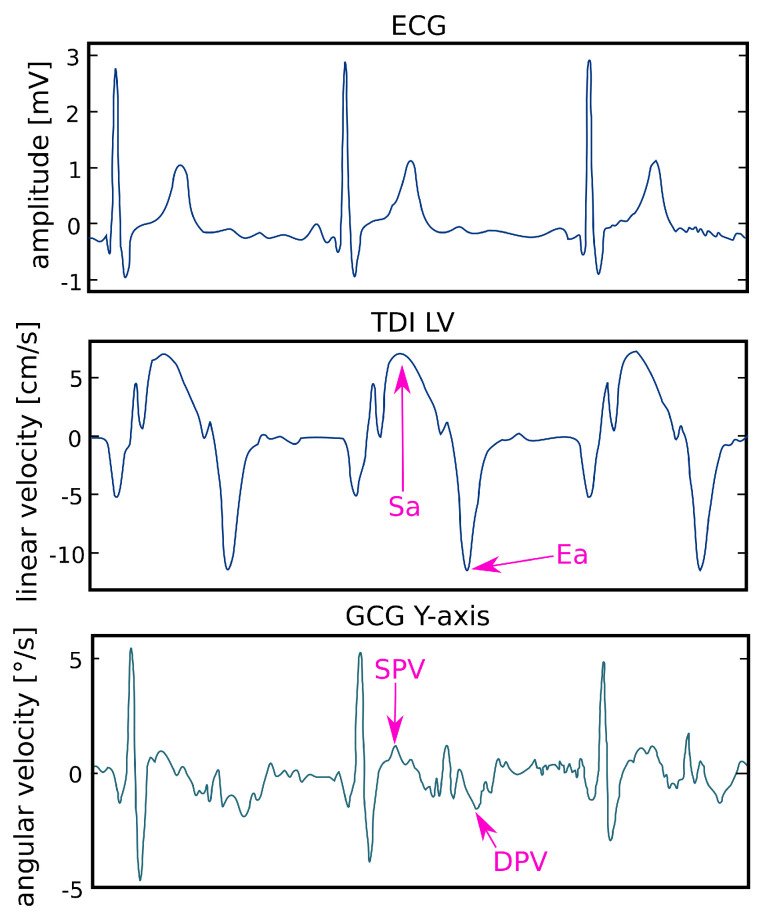
The comparison of ECG, LV TDI and GCG y-axis with marked Sa, SPV, Ea and DPV. An excerpt of a diagram in the article by Tadi et al. [11] under the CC-BY 4.0 license.

Gyrocardiographic waveform reflects also systolic velocity denoted in Figure 7 as SPV (systolic peak velocity) and diastolic velocity denoted as DPV (Diastolic peak velocity). Based on the observation in [11], SPV and DPV are strongly correlated with Sa (Systolic myocardial velocity) and Ea (early diastolic velocity) in TDI, which measure longitudinal systolic function and diastolic function [11].

**Figure 8:**
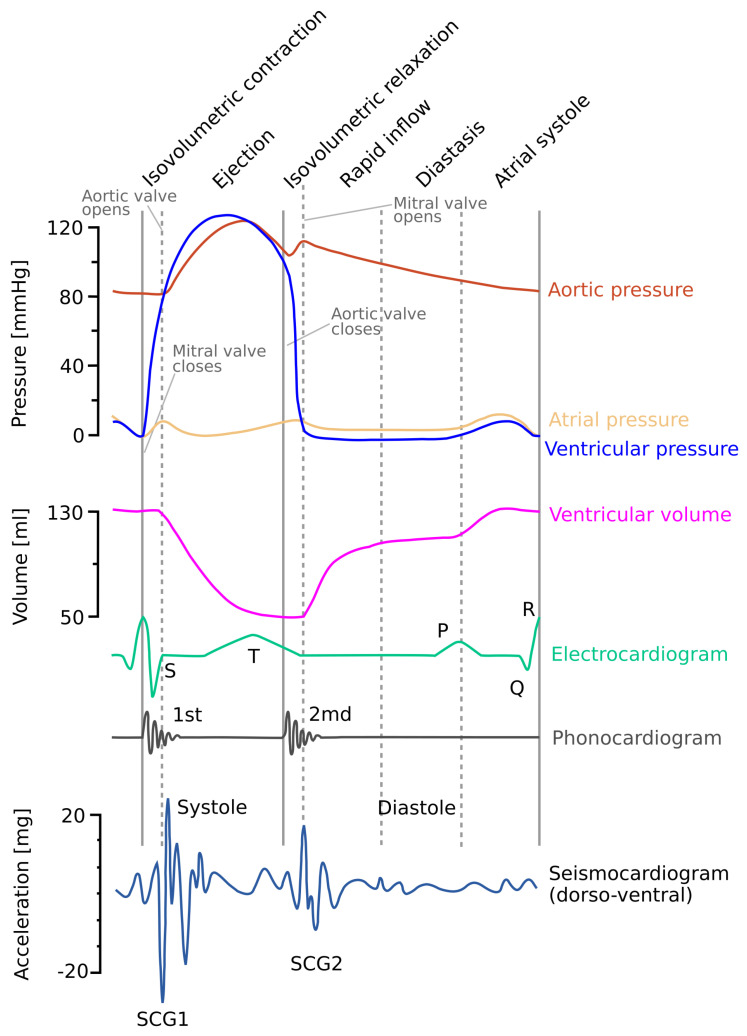
Modified Wiggers diagram. A sample of the SCG signal in the dorso-ventral (z) axis is shown alongside other cardiovascular signals: the aortic pressure, atrial pressure, ventricular volume, electrocardiogram, and phonocardiogram. The mitral valve closure (MC) and opening (MO), and aortic valve closure (AC) and opening (AO) are labeled based on the pressure signals. Modified version of the diagram presented in the article by Taebi et al. [70] shared under the license CC-BY 4.0.

Figure 8 shows a modified Wiggers diagram [96] where a sample SCG signal (in the dorso-ventral direction) is plotted along with aortic blood pressure, ventricular volume, and the electrocardiogram.

### 3.4. Waveform Description

The GCG signal has characteristic fiducial points related to the cardiac cycle. GCG morphology is consistent between different gyroscopes despite the differences in technical specifications (noise level, power consumption, full scale, range) [11]. Despite the increasing number of available works on gyrocardiography, there is no widely accepted consensus on the description of fiducial points. There are three available descriptions: the description based on ballistocardiography, the description based on seismocardiography, and the fiducial points marked as the combination of the axis and number of the point.

**Figure 9:**
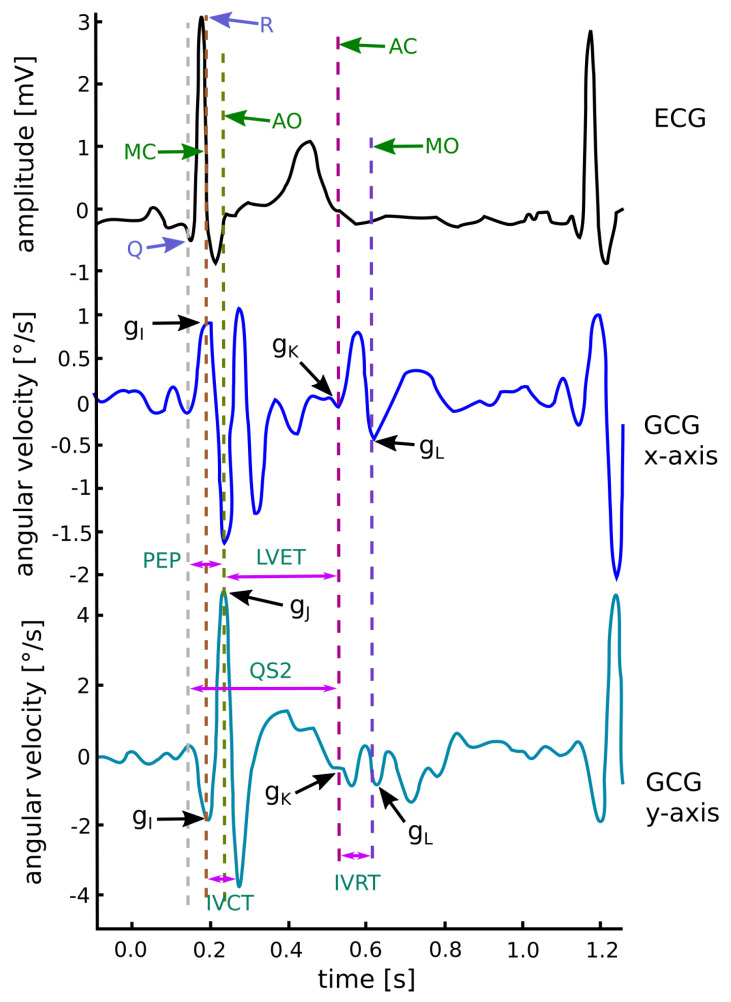
Waveform annotation in GCG x- and y-axis with corresponding time intervals and reference ECG. Modified version of the diagram by Tadi et al. published in [11] under the CC-BY 4.0 license.

The GCG fiducial points in [11] are labeled as $g_{I}$, $g_{J}$, $g_{K}$, and $g_{L}$ waves in the cardiac cycle. The $g_{I}$ wave is denoted as a fast downward notch in the y-axis around the ECG R wave. The $g_{J}$ wave is the major maximum peak in the y-axis slightly after the R wave in the ECG signal, at the moment of the opening of aortic valve [11] Because of this fact, the $g_{J}$ wave can be considered to be the occurrence of the aortic valve opening [83,84]. The $g_{K}$ wave is visible as the first notch of the lower magnitude up-down wave in the middle of the cardiac cycle, roughly after the T wave in the ECG (around the second heart sound). The second notch ot the up-down wave after the $g_{K}$ wave is defined as the $g_{L}$ wave [11]. The annotation of GCG waveforms and the corresponding cardiac time intervals with ECG reference are shown in Figure 9.

The waveform description proposed in [11] uses the convention of naming the fiducial points as the letters I, J, K and L, as in the ballistocardiogram [27]. Hence, this approach may be defined as the waveform description based on ballistocardiography.

The description of fiducial points based on seismocardiography is based on the fact that the GCG peaks correspond to the same physiological events as the SCG peaks [32,40,76,92]. Simultaneous recording of SCG and electrocardiogram (ECG) indicated that the peaks and valleys of the SCG correspond to known physiological events including mitral valve opening (MO) and closure (MC), isovolumetric contraction, ejection, aortic valve opening (AO) and closure (AC), and cardiac filling [27,95]. Tadi et al. in [11] stated that the fiducial points $g_{I}$-$g_{L}$ correspond to the opening and closing of the heart valves: $g_{I}$ corresponds to the MC wave, $g_{J}$ corresponds to the AO wave, $g_{K}$ corresponds to the AC wave and $g_{L}$ corresponds to the mitral valve opening. This approach is used in [73,83,84].

The third type of waveform description (fiducial points marked as the combination of the axis and number of the point) is described in [35]. The fiducial points (local maxima of the GCG signal) are described as X1, X2, X3 denote the first, second and third local maximum in the X component and Y1, Y2, Y3 for the first, second and third local maximum in the Y component.

#### 3.4.1. The Periods in Gyrocardiography

The analysis of periods in gyrocardiogram includes determining the isovolumetric contraction time (IVCT), isovolumetric relaxation time (IVRT), systolic time interval (STI) and the indices of cardiac contractility: total electromechanical systole (QS2), left ventricular ejection time (LVET), and the pre-ejection period (PEP) [11]. PEP and LVET play an important role in the evaluation of myocardial contractility [97,98,99].

Periods in the GCG are determined as follows: The QS2 is defined as the interval between the ECG Q wave and the AO ($g_{J}$) wave in GCG. The LVET is measured as the interval between the aortic valve opening represented in GCG as the AO wave and the aortic valve closure visible in the GCG as the AC ($g_{K}$) wave. If the simultaneous recording of the ECG is available, the pre-ejection period (PEP) is calculated as the interval between the ECG Q-wave and the onset of the aortic valve opening (AO) wave in GCG [36,97,98,99].

#### 3.4.2. Signal Morphology in Cardiac Diseases

In normal conditions, the cardiac cycle follows regular rhythm and the electrical and mechanical activity reflected in the ECG, SCG and GCG signals have monomorphic repeating patterns [11,55,77].

Reviewed works describe the GCG signal morphology in atrial fibrillation (AFib) [11,55,65,77], coronary artery disease (CAD) [55], myocardial infarction [60] and heart failure [77]. Atrial fibrillation (AFib) is a common cardiac rhythm abnormality (arrhythmia) characterized by uncoordinated atrial vibrations and ineffective atrial contraction which result in abnormal systolic-diastolic functioning [100,101,102]. In atrial fibrillation cardiac vibrations signals have irregular morphology and rhythm [55,61,65,77].

**Figure 10:**
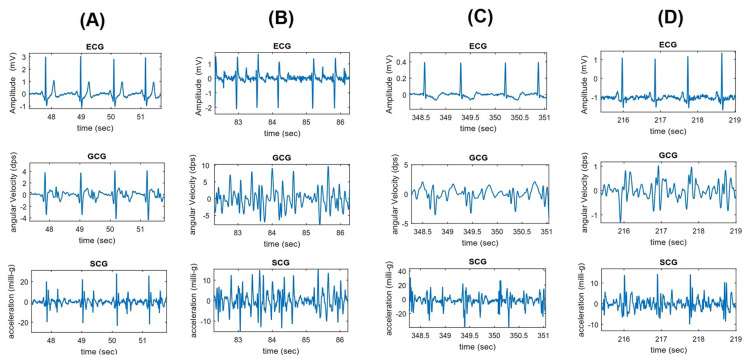
Overall ECG (lead I), GCG and SCG waveform characteristics of normal (**A**), atrial fibrillation (**B**), and coronary artery disease with ischemic changes: T-wave inversion (**C**) and ST segment depression (**D**). The work by Iftikhar et al. [55] under the CC-BY 4.0 license.

Coronary artery disease (CAD) is the reduction of blood flow to the heart muscle due to the accumulation and inflammation of plaque in coronary arteries which may cause a myocardial infarction [103,104]. In CAD the regular (sinus) rhythm is visible; however, the signal morphology has undergone considerable changes related to poor contractility (amplitude reduction), larger diastolic activity, and widened systolic complex (as in Figure 10D) where multiple and wide wavelets related to artery blockage are visible in the onset of systole [55].

**Figure 11:**
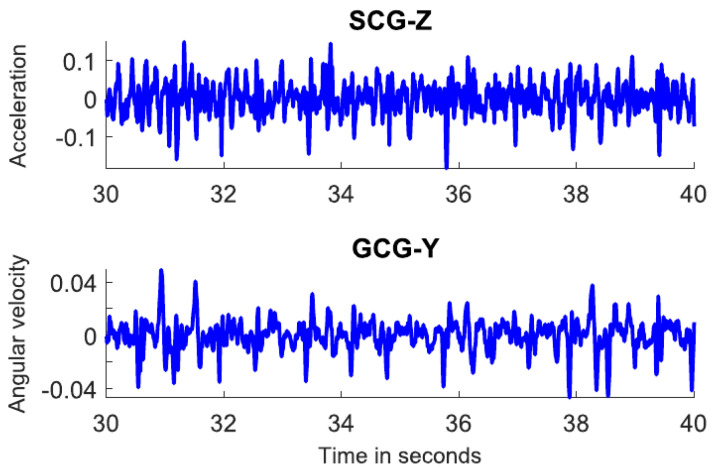
An example of AFib and acute decompensated heart failure in cardiac signals. An excerpt of the diagram published in Mehrang et al. work [77] shared under the license CC-BY 4.0.

Figure 10 shows ECG-SCG-GCG cardiac waveform characteristics in normal, AFib, and CAD conditions. The SCG and GCG waveforms in trial fibrillation combined with the acute decompensated heart failure are shown in Figure 11.

### 3.5. Applications

The number of applications for GCG is growing in recent years. The earliest applications of GCG include heart beat detection [20] and annotation of seismocardiograms [32,105]. Due to the breadth of topic and the abundance of works, the description of the applications of gyrocardiography in heart beat detection and HRV analysis are in the Section 3.5.1 and Section 3.5.2. Local pulses registered in GCG and SCG provide information that could be used for the assessment of the myocardial function and its variation, especially in the detection of cardiac arrhythmias and myocardial infarction [22,41].

Several applications of gyrocardiography are based on the fact of electromechanical delay observed in the cardiac physiology, which may bring new insights into the assessment of myocardial contractility [41]. The characteristics of cardiac vibration are correlated with physiologic [40] and pathologic [93] processes in the cardiovascular system. These applications include the quantification of beat-to-beat dynamics, analysis of cardiac intervals and hemodynamics [106]. Such information may complement other modalities, such as electrocardiography, echocardiography, cardiac MRI, serologic testing, and catheterization [70].

The detection of atrial fibrillation in gyrocardiography is based on the irregularities of inter-beat intervals [49,60,61,64,65,77,107,108]. In [108] the achieved average true positive rate (sensitivity) was 99.9% and an average true negative rate of 96.4% in leave-one-out cross-validation. The classification was based on linear least-squares classifier. The method proposed in [64] is based on singular spectrum analysis, envelope detection, zero crossing rate, time domain, entropy and spectral features and classification using KSVM with a majority vote learner and LOOCV. The accuracy of this approach was 97%.

Clairmonte et al. in [79] designed the classifier of two lung volume states (high and low volume state) on 50 participants based on 1D convolutional neural network (CNN). High volume state was defined when the subject had fully inhaled the air whereas the low volume state was when the subject had fully exhaled the air. The accuracy, precision and recall were 99.4%, 99.4% and 99.5% which indicates the feasibility of using gyrocardiography in distinguishing the state of lung volume in the cardiac cycle.

Another field of application in GCG proposed by Tadi et al. [33,46] is PET/CT (positron emission tomography/computed tomography) cardiac and respiratory gating—an approach to enhance the quality of PET/CT images by dividing the PET/CT data into individual bins that correlate with the phases of respiratory and/or cardiac motion [46,109]. The main reason of PET/CT gating are the inaccuracies in image quantification, blurring and other artifacts in cardiac and oncology imaging [110].

Yang et al. proposed classifying aortic stenosis based on the ECG, PPG, SCG and GCG signals on the patients after aortic valve replacement (TAVR) procedure [78] using the decision tree, the random forest (RF), and the artificial neural network (NN). The signals were band-pass filtered, denoised with signal energy thresholding, divided into 10-second segments and then, the continuous wavelet transform (CWT) and empirical mode decomposition (EMD) were applied. The classifier was fed with 30 features based on CWT and EMD of the GCG signal.

D’Mello et al. in [80] described a method for the identification of the heart sounds using seismocardiography and gyrocardiography. The method was based on the effective jerk and rotational kinetic energy (RKE) waveforms in study conducted on 15 subjects. The identification accuracy of the first heart sound was very high (correlation coefficients of 0.9887 for HR measured with concurrent ECG measurement).

In [60] the classification of diseases was based on machine learning, namely SVM with leave-one-out cross-validation (LOOCV) [60] and in [55] KSVM and random forests (RF) with or without majority voting was used to distinguish between myocardial infarction and coronary artery disease. Yang et al. [78] proposed classifying aortic stenosis using ANOVA test results and three types of classifiers (artificial neural networks, decision trees and random forests) on features derived from ECG, PPG, SCG and GCG signals.

Mehrang et al. in 2019 used random forest (RF), extreme gradient boosting (XGB), support vector machines (SVM), and artificial neural network (ANN). The highest sensitivity was achieved for random forests in self-applied measurement (0.948) and the highest specificity was achieved for artificial neural networks (0.936) for self-applied measurements and random forests and XGB (0.980) in the physician-applied measurements [65]. In 2020 Mehrang et al. used RF, XGB and LR and the best performance of classification between healthy subjects and patients with AFib was achieved for random forests and XGB. The best performing classifier was RF in distinguishing between healthy subjects and patients with acute decompensated heart failure and [77].

Based on the review of available works on the GCG, the overall list of applications is as follows:

- heart beat detection [9,20,37,39,48,53,57,58,81,83,84],
- fetal heart rate extraction [72],
- the analysis of hemodynamics [66], including:
  - stroke volume estimation [111],
  - IVCT, LVET [11], PEP [11,112], QS2 estimation [11].
- cardiac and respiratory gating in PET/CT [33,46,69,113],
- HRV analysis [47,65,77,83,84],
- annotation of seismocardiograms [39],
- annotation of heart sounds [80],
- diagnosing of various cardiovascular diseases, including:
  - atrial fibrillation [32,49,60,61,64,65,77,108],
  - myocardial infarction [54,55,60],
  - coronary artery disease [55],
  - classification of aortic stenosis [78],
  - heart failure [77],
- sleep monitoring [114],
- identification of heart sounds [80],
- extracting respiration wave [33,113,115],
- estimation of lung volume [73],
- cardiac monitoring of dogs [47],
- cardiac monitoring in workplace [74].

#### 3.5.1. Heart Beat Detection

Heart beat detection is one of the earliest proposed applications of gyrocardiography—the first study on this topic was conducted by Jia et al. in 2015 [20]. Although they did not name the angular velocity registered on the chest wall, we may treat the acquired signals as seismocardiograms and gyrocardiograms based on the definition in [8]. The algorithm proposed in [20] consists of wavelet-based denoising (with biorthogonal 5.5 wavelet), extracting the signal envelope with the Hilbert transform, low-pass filtering and estimation of the spectrum using a 6th order autoregressive model. The second study was conducted by Olli Lahdenoja et al. in 2016. Their approach was based on the autocorrelation in six axes (3-axis SCG and 3-axis GCG) [47].

In 2017 Yang et al. published two works which describe the heart beat detection in GCG signals: the first study describes the heart beat detector which uses band-pass filtering and finding down-up peaks [48]. In the second study, Yang et al. use band-pass filtering, calculation of the kinetic energy waveform, ensemble averaging and searching for fiducial points [39]. In the same year Tadi et al. investigated the feasibility of MEMS cardiac gating in PET/CT in [33,46,113]. Gating is used in PET/CT imaging of the heart and involves determining the occurrence of the heart beats [109].

To detect heart beats in the GCG, they used Hilbert adaptive beat identification technique (HABIT) proposed in [116] which is based on the fact that particular waveforms appear with every heart beat [11,33]. HABIT algorithm consists of band-pass filtering, noise removal, calculating the total acceleration magnitude, Hilbert transform, approximation of the heart rate signal, adaptive thresholding, and finding local maxima [113,116]. Hurnanen et al. in 2017 presented the method for heart beat detection based on gyrocardiography which uses BALANCE algorithm which consists of band-pass filtration, triangle filtration, successive mean quantization transform (SMQT), median filtration and finding local maxima within 0.4 s [49].

In 2018 Lahdenoja et al. detected heart beats based on short-term autocorrelation on 2.5 s windows with the 1.5 s overlap in 10 s segments [61]. Z. Iftikhar et al. [55] use the same approach as in [61] to detect heart beats. In Ref [37], Lee et al. proposed the heart beat detector which consists of preprocessing based on Savitzky-Golay filter L2-normalization and ensemble averaging and the dominant frequency of the Sparse Fast Fourier Transform (SFFT) between 0.75 and 2.5 Hz. In the same year Hernandez and Cretu presented their approach to detect heart beats by using band-pass filtering, multiplying the signal by −1, squaring the signal with keeping the sign (see Equation (3)), adaptive thresholding and detecting local maxima with ignoring the faulty peaks (with unusual high or low slopes) [57]. Kaisti et al. published a data set with electrocardiograms, seismocardiograms and gyrocardiograms acquired on 29 healthy male volunteers at IEEE DataPort [63].

(3) $$y=sgn\left(x\right)x^{2}$$

In 2019, D’Mello et al. [9] used autocorrelated differential algorithm developed in [82] to detect the heart beats. Kaisti et al. in [58] proposed a stand-alone heart beat detection which uses axis selection, band-pass filtering, artifact removal, beat detection based on the envelope of the squared signal, k-means clustering of the detected local maxima and minima, and finally merging beat locations from four independent location streams [58]. The article is supplemented by a complimentary data set available on IEEE DataPort [63].

Tadi et al. in [64] proposed using singular spectrum analysis, envelope detection based on moving average filter, signal segmentation and short-term autocorrelation. In another study [69] they applied band-pass filtering, motion artifact removal, ICA, envelope extraction, filtering, local maxima detection and adaptive peak detection.

In 2020 Siecinski et al. proposed in [83,84] a method for detecting heart beats by finding the local maximum within 100 ms since the occurrence of the ECG R wave. This approach is an example of repurposing the method proposed for seismocardiograms in [86] and used the fact that the AO wave is the most prominent peak in GCG [61]. In the same year Ezz Aboulezz et al. [81] developed the beat detector based on Autocorrelated Differential Algorithm (ADA) based on the autocorrelation of the signal to calculate the HR [9,82]. The correlation of heart rate measured on GCG and reference HR obtained on ECG was very strong ($r^{2}=0.956$ when supine, $r^{2}=0.975$ when standing and $r^{2}=0.965$ across the entire data set) [81].

The performance of the analyzed beat detectors on GCG signals is presented in Table 2 as provided by the authors.

The results shown in Table 2 indicate that the heart beat detection on gyrocardiograms is reliable [83,84]. This fact is supported by the reported values of performance metrics.

Another application of gyrocardiography to estimate heart rate was presented in the study conducted by Yang et al. in 2019 on the fetal heart rate (FHR) estimation acquired by a fetal cardiotocography sensor. The next step was preprocessing which consisted of band-pass filtering, continuous wavelet transform and finally, the extraction of FHR from a fused cepstrum of registered SCG and GCG signals. Reported positive percent agreement (the equivalent of sensitivity/TPR when the reference is uncertain [75]) was 75.52% from GCG [72]. To the best of the authors’ knowledge it is the first reported approach to use GCG in FHR estimation at the time of conducting literature review.

#### 3.5.2. HRV Analysis

The first attempt to perform HRV analysis on GCG signals was the study by Lahdenoja et al. in [47]. The next study which involved the HRV analysis on GCG signals was performed by Tadi et al. [53] and the main purpose was the automatic assessment of signal quality. Another studies which used HRV analysis on gyrocardiograms were conducted by Iftikhar et al. [55] and Mehrang et al. [77]. Features based on the HRV analysis on gyrocardiograms (mean absolute differences of heart rate, second-order differences and absolute second-order differences of inter-beat intervals) were used to distinguish between healthy subjects and patients with atrial fibrillation, coronary artery disease and acute decompensated heart failure [55,77].

In [47] the HRV analysis was performed in time domain and frequency domain and the results of the HRV analysis were presented as the mean absolute error between the HRV indices calculated for SCG/GCG signals and the HRV indices obtained for the ECG signal (ground truth). Time domain HRV indices included the mean inter-beat interval (AVNN), standard deviation of the inter-beat interval (SDNN), root mean square of successive differences between inter-beat intervals (RMSSD), the number of successive RR intervals differing more than 50 ms (NN50), probability of NN50 against total number of inter-beat intervals (pNN50), HRV triangular index and baseline width of the RR interval histogram (TINN). Frequency domain HRV indices include the power spectrum in very low frequency range (VLF), low frequency range (LF), high frequency range (HF), and the LF/HF power ratio [47] as defined in [117,118].

HRV analysis in [53] was performed in time domain and frequency domain. Time domain indices included the AVNN, SDNN, RMSSD, mean value, and variance of the analyzed 10 s segment, and signal quality index. The HRV analysis in frequency domain was expressed as the energy in low frequency range (1–3 Hz), high frequency range (3–20 Hz) and the ratio of the energy in low frequency and high frequency range. The Ref [53].

The studies on detecting atrial fibrillation in GCG are based on the irregularities of the inter-beat intervals [61,64,65,77]. In this context, HRV analysis is a tool for the assessment of temporal randomness in the cardiac mechanical signal [108]. In [64,65] three HRV parameters (indices) proposed in [108] were used to differentiate AFib from normal (sinus) rhythm: RMSSD, median difference of successive inter-beat intervals and the spectral density of the HRV. Then, the parameters became signal features fed to SVM, RF, robust boosting-based classifiers.

In 2020 Siecinski et al. [83,84] performed the HRV analysis on gyrocardiograms. HRV analysis included time domain and frequency domain analysis based on [86], and also non-linear HRV analysis based on Poincaré maps, based on the approach in [119]. The studies confirmed strong correlation of HRV indices calculated on ECG and GCG signals, which indicate the reliability and feasibility of HRV analysis in GCG for healthy volunteers [83,84].

## 4. Discussion

In this review, we analyzed the current state of knowledge of gyrocardiography by analyzing the availabele works on this topic published since 2016. The main focus of our review was to present the history, definition of gyrocardiography, waveform description, and applications.

The development of gyrocardiography emerges from the studies on the invasive cardiac motions performed since 1975 [12] and the development of new MEMS gyroscopes [32,50]. Since 2016, the gyrocardiography is defined as a non-invasive technique for registration of rotational component of cardiac vibrations on the chest [8,11].

Recording of local pulses using accelerometers or gyroscopes provides quantitative tools for the assessment of the myocardial function and its variation [22,41] due to the possibility of observing the changes in the electromechanical delay in the cardiac cycle, known as myocardial mechanical dispersion [41]. The characteristics of cardiac vibrations contain the information that correlate with cardiovascular physiologic [40] and pathologic processes [93].

GCG waveform has distinctive features which appear in every cardiac cycle and play an important role in the analysis [11]. These features are namely waves and intervals [66]. Four basic waves represent the closure and opening of mitral and aortic valves [11]; other waves represent rapid filling [27,95] and isovolumetric contraction [39].

Cardiovascular conditions have distinctive waveforms in GCG; in atrial fibrillation (AFib) the waveforms have irregular morphology and rhythm [55,61,65,77] due to the atria failure in mechanical function and abnormal systolic-diastolic function [55]. In CAD cardiac motion pattern indicates considerable changes related to poor contractility (amplitude reduction), larger diastolic activity, and widened systolic complex [55].

Wearable/mobile GCG as a promising mechanical cardiac monitoring tool may find its use in quantification of beat-by-beat dynamics of cardiac time intervals and can potentially represent information related to the hemodynamic variables and myocardial contractility [11]. Other applications include extracting the respiratory wave [33,113], PET/CT gating [33,113], classification of various cardiac diseases [55,77], cardiac monitoring of dogs [71].

The advantages of GCG over other modalities are as follows:

- Small in size, accurate and readily available sensors [32],
- Only one sensor is required to perform the registration [32],
- No need for applying multiple electrodes [9,120],
- The signal is not affected by gravity [32],
- Signal registration is insensitive to the location of sensor relative to the heart [32],
- The possibility of:
  - distinguishing the systolic and diastolic phases for analyses of left ventricular performance [80,121],
  - embedding the sensor into smart clothes [122],
  - performing the signal acquisition using sensors embedded in smartphones [64,71,123],
- Providing the opportunity to acquire respiratory movements for further analysis [69,113],
- High correlations with seismocardiograms [8], which are described since 1957 [30,92],
- Better noise rejection performance than in seismocardiograms [8,105],
- Better performance in PEP estimation than in SCG [112].

Because the ECG is considered the gold standard in analyzing cardiac activity [46], the advantages of gyrocardiography are underlined below as follows: small in size, inexpensive and accurate sensors [32] which can be embedded in smart clothes [122] and smartphones [64,71,123], no need for applying multiple electrodes which may irritate the patient’s skin [9,120], providing the information on the mechanical aspects of the cardiac cycle [8,11] and consequently the ability to distinguish the systolic and diastolic phases for analyses of left ventricular performance [80,121], providing the opportunity for registering respiratory movements [79], and the feasibility of self-application of the GCG using a smartphone [64,71].

The reason of better noise rejection performance and better performance in PEP estimation based on GCG is the fact that the rotational energy contains a significant portion (more than 60%) of the total SCG kinetic energy in both healthy subjects and heart disease patients [21,56].

Unfortunately, gyrocardiography has its limitations. GCG signals have interpersonal variations due to individual differences, e.g., in sensor placement, body mass index (BMI), age, sex, somatic and health conditions, resulting in vastly diverse beat morphologies. Moreover, gyrocardiograms (like other mechanocardiographic signals) are susceptible to motion artifacts that can easily overshadow the rhythm signal and thus affect the quality of the recording [27,28,58].

To summarize, the limitations of GCG found in the analyzed works are:

- Lack of widely accepted standard of waveform description,
- Gaps in knowledge of the relationship between the GCG waveforms and cardiac motion [11,40],
- Susceptibility to motion noise [27,28,58],
- Inter- and intra-subject variability [27,28,69],
- Lower temporal accuracy of GCG peaks than in SCG [39].

A severe limitation of the available studies is a low number of participants which mainly consist of young and healthy people (with no cardiovascular conditions) [8,11]. However, there are the studies which include patients with cardiac diseases, such as the studies which include atrial fibrillation [32,61,64,65], myocardial infarction [54,55,60], the study conducted by Kaisti et al. [58] evaluated the beat detection on gyrocardiograms on 12 patients with coronary artery disease and the study conducted by Yang et al. in 2019 on the classification of aortic stenosis on 20 patients and 20 healthy subjects [78].

More participants were considered in the study by Tadi et al. [64], which involved 435 subjects (including 190 with AFib) and the study by Mehrang et al. in 2019 was conducted on 300 subjects with atrial fibrillation and 182 among them were considered in further analyses because they were able to perform self-applied recording of GCG signals [65]. In 2020 Mehrang et al. conducted their study on 150 cardiac patients with AFib and 75 subjects with acute decompensated heart failure [77].

The diagnosis of cardiac diseases via gyrocardiograms is based on sophisticated signal processing techniques which involve time domain and frequency domain analysis [108] and classifiers, such as SVM with leave-one-out cross-validation (LOOCV) [55,60], KSVM, random forests with or without majority voting [55,65,78], artificial neural networks, decision trees [65,78], extreme gradient boosting [65], hierarchical classifiers [77], and 1D convolutional neural network (CNN) [79]. The use of sophisticated methods of signal processing is needed due to the inter-personal variability in the signal morphology and gaps in knowledge to be addressed [69,70,116].

Home monitoring proposed in many studies as a potential application of gyrocardiography [8,11,54,65,83,84,108], however, there are only two which actually were conducted in a self-applied manner which indicate the potential use in home monitoring. The study conducted by Mehrang et al. [65] on detecting AFib consisted on twice registration of GCG signal in clinical conditions: the first one applied by a physician and the second one which was self-applied by patients. The study on cardiac activity of dogs described in [71] also uses two stages of signal acquisition: in clinical conditions and at home.

Future studies should concentrate on addressing the limitations of registration and analysis of GCG signals, establishing a standard of signal annotation and include more health conditions in the studies in order to provide reference values. A few problems reported in [27] to be addressed are: the mapping of each measurement modality to the other, the physiological origins of the signals and the availability of signal databases and processing tools.

## 5. Conclusions

Gyrocardiography is becoming a valuable technique for a non-invasive assessment of mechanical function of the heart. The availability of inexpensive, small and accurate MEMS gyroscopes which are embedded in consumer electronics devices such as smartphones may provide new opportunities in the development of home monitoring of the heart. However, the most important limitations are the gaps in the knowledge about the relationship between the GCG waveforms and the cardiac motion [11,40], the need for standardization, susceptibility to motion noise [27,28,58], inter- and intra-subject variability in the signal morphology [27,28,69] and lower temporal accuracy of GCG peaks than in SCG [39].

The problems to be addressed in future studies on GCG are closely related to the problems of other cardiac mechanical signals. As suggested by [27], the problems to be addressed are: the mapping of each measurement modality to the other, the physiological origins of the signals and the availability of signal databases and processing tools.

## Figures and Tables

**Table 1:** Number of works on gyrocardiography per year in various sources on 1 October 2020.

Database/Search Engine	Number of Articles					
	Number of Articles Per Year					TOTAL
	2016	2017	2018	2019	2020	
Google Scholar	4	12	26	34	31	107
Web of Science Core Collection	2	5	5	5	3	20
Scopus	2	6	5	7	6	26
IEEEXplore	2	3	5	5	4	19
PubMed	1	2	5	6	3	17
Springer Link	0	0	1	2	0	3

**Table 2:** The comparison of the performance of heart beat detection in GCG signals.

Authors	Year	Reference	Performance Metrics
Tadi et al.	2017	[113]	TPR ^1^: 99.6%; PPV ^2^: 99.8%
Yang et al.	2017	[39]	Accuracy: 96.8%
Hurnanen et al.	2017	[49]	Average missed peaks: 0.22%
			False positive peaks: 0.21%
			Mean errors: 0.47%
Lee et al.	2018	[37]	$r^{2}=0.948$ (standing, relaxed)
			$r^{2}=0.725$ (sitting, relaxed)
			$r^{2}=0.988$ (standing, aroused)
			$r^{2}=0.940$ (sitting, aroused)
Hernandez and Cretu	2018	[57]	Mean absolute error: −0.3505 BPM ^3^
			Standard deviation of the absolute error: ± 2.7167 BPM
Kaisti et al.	2019	[58]	TPR: 99.9% for healthy subjects and 95.9% for heart disease patients
			PPV: 99.6% for healthy subjects and for 95.3% for heart disease patients
Tadi et al.	2019	[69]	TPR (Mean ± SD ^4^): 0.94 ± 0.06
			PPV (Mean ± SD): 0.93 ± 0.08
			F1 (Mean ± SD): 0.93 ± 0.06
D’Mello et al.	2019	[9]	TPR: 0.9657 (96.57%)
			PPV: 0.9968 (99.68%)
Aboulezz et al.	2020	[81]	$r^{2}=0.956$ when supine
			$r^{2}=0.975$ when standing
			$r^{2}=0.965$ across the entire data set

^1^ True positive rate. ^2^ Positive predictive value. ^3^ Beats per minute. ^4^ Standard deviation.

## Abbreviations

The following abbreviations are used in this manuscript:

°/s	degrees per second
1D	one-dimensional
3D	three-dimensional
AC	Aortic valve closure
ADA	Autocorrelated Differential Algorithm
AFib	Atrial fibrillation
ANN	Artificial Neural Network
AO	Aortic valve opening
AVNN	mean inter-beat interval
BCG	ballistocardiography
BMI	Body Mass Index
BPM	Beats per minute
CAD	Coronary artery disease
CNN	Convolutional neural network
CWT	Continuous Wavelet Transform
CT	Computed Tomography
dps	degrees per second
DPV	Diastolic peak velocity
Ea	Early diastolic velocity
ECG	Electrocardiography
EMD	Empirical Mode Decomposition
FHR	Fetal Heart Rate
GCG	Gyrocardiography
HABIT	Hilbert adaptive beat identification technique
HF	the power of the HRV spectrum in the high frequency range
HR	Heart rate
HRV	Heart rate variability
ICA	Independent Component Analysis
IMU	Inertial Measurement Unit
IVCT	Isovolumetric contraction time
IVRT	Isovolumetric relaxation time
KSVM	Kernel Support Vector Machine
LBP	Local Binary Patterns
LOOCV	Leave-one-out Cross-Validation
LF	the power of the HRV spectrum in the low frequency range
LF/HF	LF/HF power ratio
LR	Logistic regression
LV	Left ventricular
LVET	Left Ventricular Ejection Time
MC	Mitral valve closures
MCG	Mechanocardiography
MEMS	Microelectromechanical systems
MO	Mitral valve opening
MRI	Magnetic Resonance Imaging
NN50	number of successive RR intervals differing more than 50 ms
PCG	Phonocardiogram
PEP	Pre-ejection Period
PET	Positron Emission Tomography
pNN50	probability of NN50 against total number of inter-beat intervals
PWD	Pulse Wave Doppler
QS2	Total electromechanical systole
RF	Random Forest
RKE	Rotational Kinetic Energy
RMSSD	Root mean square of successive differences between inter-beat intervals
Sa	Systolic myocardial velocity
SCG	Seismocardiography
SD	Standard deviation
SDNN	Standard deviation of the inter-beat interval
SFFT	Sparse Fast Fourier Transform
SMQT	successive mean quantization transform
SPV	Systolic peak velocity
STI	Systolic time interval
SVM	Support Vector Machine
TAVR	Transcatheter aortic valve replacement
TDI	Tissue Doppler Imaging
TIMM	Baseline width of the RR interval histogram
VLF	the power of the HRV spectrum in the very low frequency range
XGB	Extreme Gradient Boosting
